# Características de la trombosis venosa cerebral en pacientes de dos hospitales universitarios de Colombia en el período 2018-2020

**DOI:** 10.7705/biomedica.6877

**Published:** 2023-06-30

**Authors:** Adriana Marcela Ruiz, Gabriel Esteban Acelas, Hernán Mauricio Patiño, Jean Paul Vergara, Miguel Arturo Silva, María Daniela Camargo

**Affiliations:** 1 Sección de Neurología, Neuromédica, Medellín, Colombia Sección de Neurología Neuromédica Medellín Colombia; 2 Sección Neurología, Hospital de San José, Fundación Universitaria de Ciencias de la Salud, Bogotá, D.C., Colombia Sección Neurología Hospital de San José Fundación Universitaria de Ciencias de la Salud Bogotá, D.C. Colombia; 3 Sección Neurología, Hospital Universitario San José Infantil, Bogotá, D. C., Colombia Universidad de Antioquia Sección Neurología Hospital Universitario San José Infantil Bogotá, D. C. Colombia

**Keywords:** trombosis de los senos intracraneales, factores de riesgo, cefalea, trombosis venosa, hemorragias intracraneales, Sinus thrombosis, intracranial, risk factors, headache, venous thrombosis, intracranial hemorrhages

## Abstract

**Introducción.:**

La trombosis venosa cerebral es una causa infrecuente de enfermedad cerebrovascular que viene en aumento a nivel mundial. A pesar de ello, actualmente, en Colombia no se cuenta con estudios suficientes que nos permitan caracterizar epidemiológicamente la enfermedad en nuestra población para identificar los factores de riesgo y las complicaciones más frecuentes en nuestro medio.

**Objetivo.:**

Describir las características clínicas, demográficas y radiológicas, y los factores de riesgo de una serie de pacientes con trombosis venosa cerebral de dos hospitales de Colombia.

**Materiales y métodos.:**

Es un estudio descriptivo retrospectivo de pacientes hospitalizados, atendidos en el servicio de neurología de dos hospitales de Bogotá desde diciembre de 2018 hasta diciembre del 2020.

**Resultados.:**

Se incluyeron 33 pacientes. Las frecuencias más altas correspondieron a mujeres en edad fértil, en puerperio (n=7; 33,3 %) y pacientes con patologías autoinmunes (n=10; 30,3 %). El síntoma inicial más común fue la cefalea (n=31; 93,9 %), seguido de focalización neurológica (n=9; 27,2%) y crisis epiléptica (n=8; 24,2 %). El 51 % (n=17) de los pacientes tuvo un examen físico normal. El infarto venoso cerebral se presentó en el 21,1 % (n=7), la hemorragia subaracnoidea en el 12,1 % (n=4) y el hematoma intraparenquimatoso en el 9 % (n=3) del total de pacientes. El 60,6 % (n=20) quedó con nivel independiente en la escala funcional de Barthel. Ningún paciente falleció.

**Conclusiones.:**

Se encontraron características sociodemográficas, clínicas y radiológicas similares a lo reportado en la literatura mundial. Con respecto a las diferencias, se encontró en nuestro estudio compromiso de la circulación venosa cerebral profunda en un porcentaje ligeramente mayor a lo descrito, pero sin aumento de complicaciones, ni mortalidad.

## Abstract (en)

**Introduction.:**

Cerebral venous thrombosis is an uncommon cause of cerebrovascular disease, which has been increasing worldwide. In Colombia, there are not enough recent studies that allow us to determine epidemiological characteristics of the disease in our population to identify more frequent risk factors and complications according to our living conditions.

**Objective.:**

To describe clinical, demographic, and radiographic characteristics, and risk factors in a cohort of patients with cerebral venous thrombosis attended at two hospitals in Colombia.

**Materials and methods.:**

Retrospective descriptive study with patients treated in the hospitalization neurology service of two hospitals in Bogotá, Colombia from December 2018 to December 2020.

**Results.:**

Thirty-three patients were included. There was a higher incidence of cerebral venous thrombosis in women of childbearing age in the puerperium (n=7; 33.3%) and associated with autoimmune diseases (n=10; 30.3%). The most common initial symptom was headache (n=31; 93.9 %), followed by neurological focal signs (n=9; 27.2%) and seizures (n=8; 24.2%). Fifty-one percent (n=17) of the patients had a normal physical examination. Cerebral venous infarction occurred in 21.1 % (n=7), subarachnoid hemorrhage in 12.1 % (n=4), and intraparenchymal hematoma in 9 % (n=3) of all the patients. Sixty-point six percent (n=20) of the patients had a total independent Barthel functional scale. None of those died.

**Conclusions.:**

We found similar sociodemographic, clinical, and radiography characteristics to those reported in the world literature. Regarding the differences, deep cerebral venous circulation was higher than that described in previous studies but without complications increase or mortality.

La trombosis venosa cerebral se define como la formación de un trombo que ocluye las estructuras venosas intracraneales. Se presenta con mayor frecuencia en mujeres entre los 20 y los 50 años y se ha asociado a una variedad de factores de riesgo incluidos los estados protrombóticos, el uso de anticonceptivos orales, el embarazo, el puerperio, las infecciones y algunos factores mecánicos ^1^.

Se considera una causa infrecuente de enfermedad cerebrovascular que se presenta entre el 0,5 y el 1 % de la población adulta. Sin embargo, se ha documentado que tanto su incidencia como su prevalencia está en aumento mundialmente ^2^^,^^3^. Esto puede explicarse por el acceso a los nuevos métodos diagnósticos y el desarrollo de técnicas más especializadas tales como la resonancia magnética cerebral ^4^^,^^5^.

A pesar de ello, actualmente, en Colombia no hay estudios suficientes que nos permitan caracterizar epidemiológicamente la enfermedad en nuestra población, para una correcta estratificación de la presentación clínica y diagnóstico temprano, con el fin de planificar estudios futuros de prevención con datos propios de factores de riesgo y complicaciones de la enfermedad en nuestro país.

Por todo lo anterior, el objetivo de este estudio fue determinar las características clínicas, radiológicas y factores de riesgo de pacientes con diagnóstico de trombosis venosa cerebral en dos hospitales universitarios de Bogotá entre los años 2018 y 2020, por ser de referencia de enfermedad vascular a nivel nacional.

## Materiales y métodos

Se diseñó un estudio observacional retrospectivo de tipo serie de casos, en el que se revisaron las historias clínicas de los pacientes en la base de datos de hospitalización del Servicio de Neurología del Hospital de San José y del Hospital Infantil Universitario de San José. Se incluyeron todos los pacientes que cumplían los criterios de elegibilidad entre diciembre del 2018 y diciembre del 2020.

Los criterios de elegibilidad fueron: pacientes de edad mayor o igual a 18 años con diagnóstico de trombosis venosa cerebral confirmada por angiorresonancia venosa cerebral, angiografía cerebral por tomografía computarizada o arteriografía cerebral, que hayan ingresado al servicio de hospitalización de alguno de los dos hospitales.

Se recolectaron los siguientes datos: edad, sexo, síntomas de consulta, estancia hospitalaria, estancia en unidad de cuidados intensivos, factores de riesgo, examen neurológico, química sanguínea, perfil autoinmune, perfil de trombofilias, marcadores tumorales, líquido cefalorraquídeo, características radiológicas, complicaciones y escala de Barthel al final de la hospitalización.

La distribución de los datos por variable se analizó con la prueba de Shapiro-Wilk. La descripción de las características clínicas y demográficas de la serie de participantes se realizó por medio de frecuencias absolutas y relativas para las variables cualitativas, y por medio de mediana y rango intercuartílico para las variables cuantitativas. El análisis de los datos se llevó a cabo con el programa Stata 15™.

El presente trabajo fue aprobado por el comité de ética de los dos hospitales involucrados.

## Resultados

Treinta y tres pacientes cumplieron los criterios de inclusión, con una mediana de edad de 33 años [rango intercuartílico (RIC): 24-48]; fue más común en mujeres (n=27; 81,8 %), de las cuales el 78 % (n=21) se encontraban en edad reproductiva (18-49 años) (cuadro 1).

**Cuadro 1:** Características sociodemográficas y síntomas de consulta

Características sociodemográficas		% (n)
Edad (años)		Mediana de edad: 33
		RIC: 24-48
Sexo		Masculino: 18,2 (6/33)
		Femenino: 81,8 (27/33)
Síntomas de consulta		% (n)
Cefalea		93,9 (31/33)
	Único síntoma	36,3 (12/33)
Focalización neurológica		27,2 (9/33)
Crisis epiléptica		24,2 (8/33)
Alteración del lenguaje		18,2 (6/33)
Alteración del contenido de conciencia		12,1 (4/33)
Compromiso de pares craneales		12,1 (4/33)
Alteración de estado de conciencia		9,1 (3/33)
Alteración de agudeza visual		6 (2/33)
Fiebre		3 (1/33)
Examen neurológico		
Normal		51,5 (17/33)
Estado de conciencia, alerta		100 (33/33)
Alteración del contenido de la conciencia		6 (2/33)
Compromiso del lenguaje y/o del habla		12,1 (4/33)
Compromiso de pares craneales		21,2 (7/33)
Compromiso motor		18,1 (6/33)
Alteración sensitiva		15,1 (5/33)
Alteración de la marcha		6 (2/33)
Alteración de la coordinación		6 (2/33)

Se identificaron factores de riesgo en el 81 % (n=27) de los pacientes. Del total de la población se encontró con mayor frecuencia el antecedente de enfermedades autoinmunes en un 30,3 % (n=33), siendo hipotiroidismo (n=5; 15,1 %) la más común, seguida del síndrome de anticuerpos antifosfolipídicos (n 3; 9,1 %) y lupus eritematoso sistémico (n=2; 6 %). En mujeres en edad fértil se determinó el embarazo como factor en el 14,3 % (n=3), el puerperio en el 33,3 % (n=7) y uso de anticonceptivos orales en el 25,9 % (n=7) del total de mujeres.

Entre otros factores temporales de riesgo en todos los participantes, hombres y mujeres, se encontró: anemia en el 21,2 % (n=7), obesidad en el 18,2 % (n=6), tabaquismo en el 15,1 % (n=5), antecedentes de cáncer en el 12,1 % (n=4) e infección del sistema nervioso central en el 9,1 % (n=3). Teniendo en cuenta la pandemia por el virus SARS-CoV-2, se documentó un solo paciente con la infección activa asociada a trombosis venosa cerebral (cuadro 2).

**Cuadro 2:** Factores de riesgo

Factores de riesgo temporales	% (n)
Anticonceptivos orales	25,9 (7/27)
Puerperio	33,3 (7/21)
Embarazo	14,3 (3/21)
Obesidad	18,2 (6/33)
Tabaquismo	15,1 (5/33)
Infección del sistema nervioso central	9,1 (3/33)
Anemia	21,2 (7/33)
Fístula dural	3 (1/33)
Hipertensión intracraneal espontánea	3 (1/33)
Antecedente de cáncer	12,1 (4/33)
Infección por SARS CoV-2 activa	3 (1/33)
Trauma craneoencefálico	0 (0/33)
Factores de riesgo definitivos	
Antecedente de enfermedad autoinmune	30,3 (10/33)
Deficiencia de antitrombina III	9,09 (1/11)
Deficiencia de proteína C	9,09 (1/11)

En los pacientes hospitalizados se obtuvo el perfil de trombofilias al 33,3 % (n=11). Sólo en un paciente se encontró deficiencia de antitrombina III (9 %) y en otro, deficiencia de la proteína C de coagulación (9 %) como factor de riesgo definitivo (cuadro 3).

**Cuadro 3:** Exámenes de laboratorio

Marcadores tumorales	
No realizado: 78,7 % (26/33) Positivo: 0 % (0/7)	
Perfil de trombofilia	
Realizado: 33,3 % (11/33) Deficiencia de antitrombina III: 9,09 % (1/11) Deficiencia de proteína C: 9,09 % (1/11)	
Perfil inmunológico	
Anticuerpos antinucleares	Positivo: 9,5 % (2/21) No realizado: 36,3 % (12/33)
Anticuerpos nucleares extractables	Positivo: 0 % (0/18) No realizado 45,4 % (15/33)
Anticoagulante lúpico	Positivo: 16,6 % (2/12) No realizado: 63,6 % (21/33)
Anticuerpos Smith	Positivo: 16,6 % (1/6) No realizado: 81,8 % (27/33)
Anticuerpos para anticuerpos antifosfolipídicos	Positivo: 9 % (2/21) No realizado: 36,3 % (12/33)
Complemento C3/C4	Consumido: 11,1 % (2/18) No realizado: 45,4 % (15 /33)
Anticuerpos anti-ADN	Positivo: 0 % (0/13) No realizado: 60,6 % (20/33)

Entre los síntomas de presentación clínica del total de pacientes, el más común fue cefalea en el 93,9 % (n=31), seguido por focalización neurológica en el 27,2 % (n=9), crisis epilépticas en el 24,2 % (n=8) y alteración del lenguaje en el 12,1 % (n=4). La mediana de estancia hospitalaria fue de seis días (RIC=4,5-9,8 días), con estancia mínima de un día y máxima de 37 días. Sólo dos pacientes (6 %) requirieron unidad de cuidados intensivos (cuadro 1).

**Figura 1:**
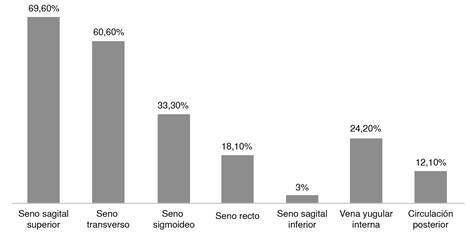
Localización de la trombosis venosa cerebral

En los estudios imagenológicos, el seno longitudinal superior fue el más afectado (n=22; 67 %), seguido del seno transverso (n=20; 60 %) y el seno sigmoideo (n=11; 33,3 %). Hubo compromiso del sistema venoso profundo en el 12,1 % (n=4) (figura 1). Identificamos la presencia de infarto venoso cerebral en el 21,2 % (n=7), hemorragia subaracnoidea en el 12,1 % (n=4) y hematoma intraparenquimatoso en el 9 % (n=3) del total de pacientes a los que se les realizó resonancia, angiorresonancia magnética cerebral o ambas (cuadro 4). El 42,8 % (n=3) de los pacientes con infarto venoso cerebral tenían compromiso de sistema venoso profundo.

**Figura 2:**
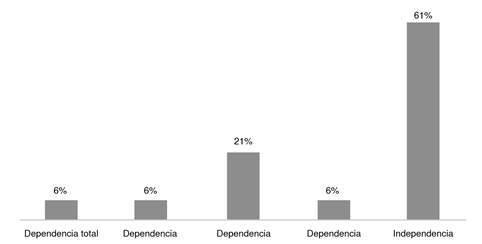
Escala de Barthel

De las complicaciones inmediatas a la hospitalización se documentó un paciente con hipertensión intracraneal y según la evaluación de escala funcional de Barthel, dos pacientes con dependencia total, dos con dependencia grave y siete con dependencia moderada (figura 2). En el presente estudio, ningún paciente falleció durante la hospitalización.

## Discusión

Basándonos en la literatura revisada, en nuestro medio son pocos los estudios que describen las características epidemiológicas de los pacientes con trombosis venosa cerebral. Sin embargo, se ha reportado una baja prevalencia de trombosis venosa cerebral (0,7 %) entre las enfermedades cerebrovasculares, según un estudio del Instituto Neurológico de Colombia ^6^.

Aunque no fue el objetivo de esta investigación evaluar la prevalencia de trombosis venosa cerebral, de acuerdo con los datos estadísticos de las dos instituciones, entre el 1° diciembre del 2018 y el 1° diciembre del 2020, el total de pacientes con enfermedad cerebrovascular fue de 884. Teniendo en cuenta esta totalidad de pacientes, la prevalencia de la trombosis venosa cerebral en la población estudiada fue del 3,7 %, lo que nos permite inferir que en nuestro medio es más frecuente esta patología neurológica. Esto se evidenció también al compararla con un estudio recientemente publicado en Argentina, en el que se estudió la enfermedad durante 30 años en 53 pacientes ^7^. Las diferencias en cuanto a población quizás se deban a que nuestro estudio fue realizado en dos hospitales que son referentes nacionales de enfermedad vascular en Colombia.

Con respecto a los datos demográficos, estos fueron similares a los reportados a nivel mundial como los de un estudio australiano, una serie de casos de Argentina, una revisión realizada en Norteamérica y otros estudios colombianos ^1^^,^^5^^-^^7^. La Trombosis venosa cerebral es más frecuente en mujeres en edad fértil y está asociado a factores de riesgo temporales como la anticoncepción hormonal y los estados procoagulantes propios del embarazo y el puerperio ^8^. Además, durante la investigación se observó con mayor frecuencia el antecedente de enfermedades autoinmunes, similar a lo descrito en el estudio local publicado en el 2012 por Amaya y colaboradores ^9^, y con cifras parecidas a las documentadas en el estudio prospectivo observacional realizado por 89 centros en 21 países, en el que se reportó 59 % de un síndrome antifosfolipídico ^10^.

En otros estudios controlados, otras condiciones asociadas a la trombosis venosa cerebral son las trombofilias, el cáncer, los trastornos inflamatorios y las alteraciones metabólicas como la obesidad ^8^, también documentadas en nuestro estudio, a pesar de que el perfil de trombofilias y la búsqueda activa paraneoplásica no se hizo en todos los pacientes.

Complementando los factores de riesgo y teniendo en cuenta la pandemia por el virus de SARS-CoV-2, que se reportó por primera vez en Colombia en marzo del 2020, se documentó un solo paciente con la infección activa asociada a trombosis venosa cerebral. Este caso fue publicado en la revista *Ictus,* en enero 2021 por Patiño *et al*. ^11^ y se relacionó con esta infección teniendo en cuenta que el SARS-CoV-2 produce una inflamación sistémica y una tormenta de citocinas por un mecanismo posinfeccioso directo inmunomediado. Esta respuesta está asociada a endoteliopatía y estasis y, por lo tanto, a complicaciones trombóticas y con menor frecuencia a trombosis venosa cerebral ^12^. Li y colaboradores encontraron una frecuencia del 5,9 % de enfermedad cerebrovascular aguda después de COVID y en esa cohorte, un solo paciente con trombosis venosa cerebral ^13^. Sin embargo, cuando se presenta es extensa y se asocia a mortalidad ^11^.

En relación con la presentación clínica, la trombosis venosa cerebral se manifiesta como un cuadro muy variable debido a su progresión y evolución dinámica ^14^. En nuestro estudio, el síntoma de consulta más frecuente fue la cefalea como único síntoma en el 36 % de los pacientes, como se reporta en otros estudios ^3^^,^^6^^,^^7^^,^^9^, por lo cual la trombosis venosa cerebral se debe sospechar en aquellos pacientes que manifiesten este síntoma.

Para realizar el diagnóstico de trombosis venosa cerebral se recomienda la utilización de técnicas no invasivas como la angiografía por tomografía, la resonancia magnética nuclear o ambas ^15^, siendo estas realizadas en este estudio en el 79 % y el 100 % de los casos respectivamente. Tan solo para dos pacientes nos apoyamos en la arteriografía cerebral para el diagnóstico de la trombosis venosa cerebral. Según las guías de la *American Heart Association* (AHA) / *American Stroke Association* (ASA) tan solo 30 % de los pacientes tienen anormalidades en la tomografía computarizada (TC) simple inicial de cráneo, siendo la hiperdensidad de la vena cortical el signo principal, seguido del signo delta denso e infarto cerebral con componente hemorrágico. También reportaron hemorragia subaracnoidea del 0,5 al 0,8 % ^16^, a diferencia de la proporción encontrada en nuestro estudio: el 100 % de los pacientes a los que se les realizó la TC simple de cráneo tenían signos indirectos de trombosis venosa cerebral. Documentamos infarto cerebral en el 26,9 %, hematoma intraparenquimatoso 9 % y hemorragia subaracnoidea en el 11 % de pacientes (cuadro 4). Sin embargo, estos porcentajes fueron menores al compararlos con los obtenidos en estudios locales y en un estudio internacional de cohorte multicéntrica, en donde se documentó infarto cerebral en el 36,4 % y transformación hemorrágica en el 17,3 % ^3^^,^^5^^,^^6^^,^^17^.

**Cuadro 4:** Características radiológicas

Tomografía simple de cráneo [79 % (26/33)]	% (n)	
Hiperdensidad de seno venoso	69	(18/26)
Hiperdensidad de vena cortical	23	(6/26)
Signo delta	4	(1/26)
Infarto cerebral	26,9	(7/26)
Infarto talámico	38	(3/7)
Hemorragia subaracnoidea	15	(4/26)
Hematoma intraparenquimatoso	11	(3/26)
Hallazgos en resonancia magnética cerebral y angioresonancia magnética cerebral		
Infarto cerebral en la resonancia magnética El 42,8% (n=3) tenía compromiso de sistema venoso profundo.	21,2	(7/33)
Hemorragia subaracnoidea en la resonancia magnética cerebral	12,1	(4/33)
Hematoma intraparenquimatoso en la resonancia magnética	9,09	(3/33)

En referencia a los senos venosos cerebrales en nuestro estudio, los más afectados fueron: el seno sagital superior, el seno transverso y el seno sigmoideo. Esto es similar a lo descrito en estudios locales realizados por Amaya *et al.,* Zuluaga *et al.* y Bermúdez *et al*. ^3^^,^^6^^,^^9^. Sin embargo, durante el estudio se evidenció compromiso de la circulación venosa cerebral profunda en un 12,1 % del total de los pacientes, siendo un porcentaje ligeramente mayor a lo descrito, aunque sin aumento de complicaciones, ni mortalidad.

Con respecto a las complicaciones tempranas se encontró aumento de presión intracraneana secundaria al bloqueo del flujo venoso y malabsorción de líquido cefalorraquídeo ^18^^,^^19^. En este estudio se documentó un paciente con hipertensión intracraneana, ninguno presentó hidrocefalia comunicante, probablemente por diagnóstico e inicio temprano de tratamiento (cuadro 5). Otra de las complicaciones tempranas son las crisis epilépticas descritas hasta en el 37 % de los adultos ^1^; en nuestro estudio sólo el 24 % de los pacientes presentaron crisis epilépticas como síntoma inicial. Las complicaciones tardías no se evaluaron en este estudio.

**Cuadro 5:** Complicaciones

Complicación	% (n)	
Hidrocefalia	0	(0/33)
Fístula dural	0	(0/33)
Herniación transtentorial	0	(0/33)
Hipertensión intracraneal	3	(1/33)
Muerte	0	(0/33)

En la actualidad, a pesar de la mejoría de las técnicas diagnósticas y el tratamiento oportuno, la trombosis venosa cerebral es todavía una causa frecuente de discapacidad permanente, aunque el 80 % de los pacientes presenta una adecuada recuperación con buena independencia posterior al evento trombótico ^20^. En nuestro estudio, al final de la hospitalización la mayoría de los pacientes quedaron con independencia total según la escala de Barthel. Sin embargo, no podemos escatimar los valores de dependencia moderada (21 %), total (6 %) y seria (6 %), por lo que debemos realizar un diagnóstico e inicio temprano de terapia tanto farmacológica como de rehabilitación. 

Con respecto a la mortalidad, ningún paciente falleció durante la hospitalización (cuadro 5), diferente a lo documentado en estudios internacionales en los que, aproximadamente, del 3 al 15 % de los pacientes mueren durante la fase aguda de la enfermedad ^10^^,^^16^^,^^21^^,^^22^. Es probable que el inicio temprano de la anticoagulación y una menor cantidad de complicaciones tempranas en nuestro estudio favorezca la supervivencia de los pacientes. No obstante, es necesario tener en cuenta que en nuestros centros no se realizó seguimiento posterior al egreso.

Con este trabajo concluimos que las características sociodemográficas, clínicas y radiológicas de nuestro estudio son similares a las de las cohortes locales e internacionales de pacientes con trombosis venosa cerebral. A diferencia de los otros estudios, en el análisis realizado se comprometió la circulación venosa cerebral profunda en un porcentaje ligeramente mayor a lo descrito, pero sin aumento de complicaciones, ni mortalidad.

En resumen, la trombosis venosa cerebral afecta con mayor frecuencia a mujeres en edad fértil, a pacientes que usan anticonceptivos orales y a aquellos que presentan enfermedades autoinmunes de base. Estas condiciones requieren un alto nivel de sospecha para el diagnóstico de trombosis venosa cerebral en la población de nuestros hospitales junto con la cefalea migrañosa ya que es el síntoma más frecuente.

La trombosis venosa cerebral es aún una enfermedad poco común. No obstante, su incidencia va en aumento, por lo que su estudio es esencial para conocer factores de riesgo, comportamiento en la fase aguda, respuesta al tratamiento y complicaciones en la población. Esto servirá para establecer medidas de prevención y de diagnóstico temprano con el fin de evitar secuelas neurológicas permanentes en nuestros pacientes. A futuro, podrían plantearse estudios multicéntricos en los que se incluyan pacientes con más años de observación en fase aguda y seguimiento, logrando un tamaño de muestra mayor que nos permita obtener este conocimiento en Colombia.

Para finalizar, después de la pandemia es importante tener en cuenta la infección por SARS-CoV-2 en pacientes con diagnóstico de trombosis venosa cerebral que presenten síntomas sugestivos, pues la infección por este virus se relaciona a un peor desenlace de la enfermedad por compromiso del sistema venoso profundo.
